# Evaluating the performance of the Alere PBP2a SA Culture Colony Test with the Vitek 2 Antimicrobial Susceptibility Test Card System as reference standard in coagulase-negative *Staphylococcus* species

**DOI:** 10.1016/j.imj.2024.100126

**Published:** 2024-08-06

**Authors:** Tze Shien Lo, Michihiko Goto, Kimberly D.P. Hammer

**Affiliations:** a Fargo VA Health Care System, 2101 Elm Street, Fargo, ND 58102, USA; b University of North Dakota School of Medicine & Health Sciences, 1919 Elm Street, Fargo, ND 58102, USA; c Iowa City VA Health Care System, 601 Highway 6 West, Iowa City, IA 52246, USA; d University of Iowa Carver College of Medicine, 451 Newton Road, Iowa City, IA 52242, USA

**Keywords:** PBP2a IC assay, *Staphylococcus epidermidis*, Non-epidermidis coagulase-negative *Staphylococcus*

## Abstract

**Background:**

The Alere PBP2a SA Culture Colony Test is an FDA-cleared *in vitro* immunochromatographic assay for rapid detection of penicillin-binding protein2a (PBP2a) in *Staphylococcus aureus*.

**Methods:**

We investigated the performance of the PBP2a SA Culture Colony Test with 78 coagulase-negative *Staphylococcus* (CoNS) isolates from different body sites, with the Vitek 2 Antimicrobial Susceptibility Test (AST) as a reference standard.

**Results:**

The CoNS species were 62 *S. epidermidis*; 6 *S. lugdenensis*; 3 *S. hominis*; 2 *S. capitis*; 2 *S. haemolyticus*; and 1 each of *S. simulans, S. auricularis,* and *S. warneri*. Of the 78 CoNS isolates, 68 showed concordance in the PBP2a IC assay and Vitek 2 AST. Discordance was seen for 10 *S. epidermidis* isolates, which showed negative in the PBP2a assay, despite oxacillin-resistance detection using the Vitek 2 AST (66.7% sensitivity and 100% specificity). All non-*S. epidermidis* CoNS were identified with 100% concordance using the PBP2a IC assay and Vitek 2 AST.

**Conclusion:**

We demonstrated that, while the PBP2a IC assay has low sensitivity in determining the susceptibility of *S. epidermidis* to oxacillin, it highly accurately predicted the susceptibility of non-*S. epidermidis* CoNS to oxacillin. The diagnostic accuracy for non-*S. epidermidis* CoNS needs further assessment with more isolates to confirm our findings.

## Highlights

• The PBP2a immunochromatographic (IC) assay was 66.7% sensitive and 100% specific for Staphylococcus epidermidis compared to microdilution minimum inhibitory concentration method.
• The PBP2a assay is 100% sensitive and specific in predicting the susceptibility of non-epidermidis CoNS.
• A larger number of isolates is needed to confirm our findings of high accuracy in predicting the susceptibility of non-epidermidis CoNS by PBP2a IC assay.

## Graphical abstract

**Figure:**
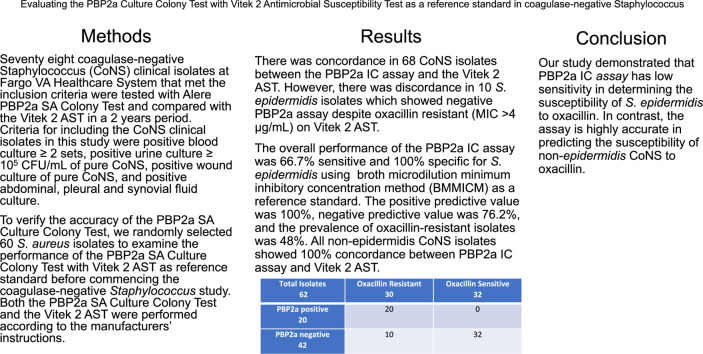

## 1 Background

The main hallmark of methicillin-resistant *Staphylococcus aureus* (MRSA) is the possession of the *mecA* gene, which produces penicillin-binding protein 2a (PBP2a). This confers resistance to beta-lactam agents, except for the fifth-generation cephalosporins ceftaroline and ceftobiprole.

The Alere PBP2a SA Culture Colony Test (Alere Scarborough, Inc., Scarborough, ME, USA) is an FDA-cleared, simple, qualitative, *in vitro* immunochromatographic (IC) assay that uses monoclonal antibodies for the rapid detection of PBP2a in *S. aureus* isolates. The test uses very sensitive recombinant monoclonal antibody fragments (rFabs) to detect the PBP2a protein from *S. aureus*. The clinical performance of the PBP2a IC test was compared with cefoxitin disk diffusion in *S aureus* isolates and showed a sensitivity of 99.1% and specificity of 99.2% on tryptic soy agar plates [1].

Because it takes about 5 minutes to identify whether an *S. aureus* isolate is MRSA without using an expensive high-tech machine, Fargo VA Healthcare System, an 80-bed Veterans Affairs Hospital in Fargo, North Dakota, has been using the PBP 2a IC test to rapidly identify MRSA from *S. aureus* clinical isolates. This has routinely been confirmed with the Vitek 2 Antimicrobial Susceptibility Test (AST) reference standard since August 2011.

## 2 Methods

Most coagulase-negative *Staphylococcus* (CoNS) species also possess the *mecA* gene; however, only a few studies have investigated the diagnostic performance of IC assays that target the PBP2a of CoNS [2,3]. The aim of this study was to validate the performance of the PBP2a IC test for clinically significant CoNS isolates using the Vitek 2 AST Card System (BioMérieuex, Inc., Durham, NC, USA) based on the broth microdilution minimum inhibitory concentration method (BMMICM) as a reference standard.

Eighty clinical CoNS isolates at Fargo VA Healthcare System that met the inclusion criteria were tested with the Alere PBP2a SA Colony Test, and the results were compared with those of Vitek 2 AST, from October 2014 to September 2016. The criteria for including CoNS clinical isolates in this study were positive blood culture of ≥ 2 sets; positive urine culture of ≥ 10^5^ CFU/mL of pure CoNS; positive wound culture of pure CoNS; and positive abdominal, pleural, and synovial fluid culture. These isolates were included because they were considered clinically significant based on American Society for Microbiology (ASM) recommendations [4]. Exclusion criteria were a positive blood culture of only one set, positive urine culture of <10^5^ CFU/mL, and positive urine culture of ≥ 10^5^ CFU/mL, but not pure CoNS or wound cultures that grew a CoNS isolate as one of the polymicrobial flora.

To verify the accuracy and examine the performance of the PBP2a SA Culture Colony Test, we randomly selected 60 *S. aureus* isolates, with Vitek 2 AST as the reference standard, before commencing the coagulase-negative *Staphylococcus* study. Both the PBP2a SA Culture Colony Test and Vitek 2 AST were performed according to the manufacturers’ instructions.

This study was approved by the University of South Dakota's Institutional Review Board and the Fargo VA Health Care System's Research and Development committee.

## 3 Results

The 60 *S. aureus* isolates selected randomly as controls demonstrated 100% concordance between the PBP2a IC assay and Vitek 2 AST.

Eighty CoNS isolates from 80 unique patients were studied, and 2 samples were excluded owing to the failure of the Vitek 2 machine to perform the oxacillin minimal inhibitory concentration (MIC) test. *Staphylococcus epidermidis* accounted for 80% of the 78 CoNS isolates that were included for analysis (*n* = 62), followed by *S. lugdenensis* (*n* = 6), *S. hominis* (*n* = 3), *S. capitis* (*n* = 2), *S. haemolyticus* (*n* = 2), *S. simulans* (*n* = 1), *S. auricularis* (*n* = 1), and *S. warneri* (*n* = 1).

A breakdown of the sources and species of the 78 coagulase-negative *Staphylococcus* isolates is shown in Table 1. Because the primary objective of this study was to solely investigate the performance of the PBP2a SA Culture Colony Test for CoNS isolates, we did not look into the clinical implications of our microbiological findings.

**Table 1:** Sources and species of the 78 coagulase-negative *Staphylococcus* isolates.

Source	Species	Number of samples
Urine	*S. epidermidis*	44
	*S. hominis*	2
	*S. lugdunensis*	1
	*S. haemolyticus*	1
	*S. warneri*	1
Wound	*S. epidermidis*	15
	*S. lugdunensis*	4
	*S. auricularis*	1
	*S. simulans*	1
	*S. capitis*	1
Blood	*S. capitis*	1
	*S. haemolyticus*	1
	*S. hominis*	1
Synovial fluid	*S. epidermidis*	1
	*S. lugdunensis*	1
Pleural fluid	*S. epidermidis*	1
Abdominal fluid	*S. epidermidis*	1

There was concordance for 68 CoNS isolates between the PBP2a IC assay and the Vitek 2 AST. However, there was discordance for 10 *S. epidermidis* isolates, which showed as negative on the PBP2a assay, despite oxacillin resistance (MIC >4 µg/mL) showing on the Vitek 2 AST. A breakdown of the sources of the 10 *S. epidermidis* isolates is as follows: 7 from urine, 2 from wounds, and 1 from abdominal fluid. There was 100% concordance between the PBP2a IC assay and Vitek 2 AST for all non-*epidermidis* CoNS isolates.

The overall performance of the PBP2a IC assay was 66.7% sensitivity and 100% specificity for *S. epidermidis* when using the broth microdilution minimum inhibitory concentration method (BMMICM) as a reference standard (Table 2). The positive predictive value was 100%, the negative predictive value was 76.2%, and the prevalence of oxacillin-resistant isolates was 48%.

**Table 2:** 2×2 table of PBP2a and oxacillin results for the 62 *Staphylococcus epidermidis* isolates.

Total Isolates 62	Oxacillin Resistant 30	Oxacillin Sensitive 32
PBP2a positive 20	20	0
PBP2a negative 42	10	32

## 4 Discussion and conclusion

Our study showed that the concordance rate between the PBP2a IC assay and BMMICM was 100% for *Staphylococcus aureus* and non-*S. epidermidis* coagulase-negative *Staphylococcus*. However, the IC assay failed to detect oxacillin resistance in 10 (33%) of the *S. epidermidis* isolates that showed oxacillin resistance by BMMICM. Two studies, one conducted in Japan and the other in France, found that an immunochromatographic assay for detecting PBP2a among non-*Staphylococcus aureus* isolates was 100% accurate. The smaller sample numbers of coagulase-negative *Staphylococcus* isolates in Matsui et al.’s and Mantion et al.’s studies (*n* = 53 and 25, respectively) might explain why both studies were unable to detect any difference between the two methodologies [5,6]. Another study conducted by Arnold and colleagues found that the Alere PBP2a culture colony test was sensitive and specific for non-*aureus Staphylococcus* species, but the studied isolates did not include *S. epidermidis* [7].

A recent study demonstrated that the methicillin-resistant isolates of CoNS that initially showed as negative in the IC assay could be induced to show as positive by harvesting colonies from around the cefoxitin disk (cefoxitin induction) [3]. We did not perform cefoxitin induction in our study and that might explain the high negative PBP2a rate among the oxacillin-resistant isolates. Another plausible explanation for the discordance between the PBP2a IC assay and BMMICM in our study is the heterogenous expression of the *mecA* gene by various species of CoNS [8,9]. Suzuki and co-workers showed that CoNS can manifest oxacillin resistance via mechanisms other than the production of PBP2a [10].

In conclusion, our study demonstrated that the PBP2a IC assay has low sensitivity in determining the susceptibility of *S. epidermidis* to oxacillin. In contrast, the assay is highly accurate in predicting the susceptibility of non-*epidermidis* CoNS to oxacillin. However, because of the small sample size, the diagnostic accuracy for non-*epidermidis* CoNS needs further assessment with a larger number of isolates to confirm our findings.

This study was the result of work performed at the Fargo VA Health Care System and supported with resources and the use of the Health Care System's facilities. The contents do not represent the views of the U.S. Department of Veterans Affairs or the United States Government.

## CRediT authorship contribution statement

**Tze Shien Lo:** Conceptualization, Data curation, Formal analysis, Writing – original draft, Writing – review & editing. **Michihiko Goto:** Formal analysis, Writing – original draft, Writing – review & editing. **Kimberly D.P. Hammer:** Formal analysis, Writing – original draft, Writing – review & editing.
